# Stakeholder Perspectives on Affinity Domains in Digital Health Interoperability: Qualitative Study

**DOI:** 10.2196/83894

**Published:** 2026-04-02

**Authors:** Jan Bruthans, Petra Hospodková, Ilona Fejfarová

**Affiliations:** 1 Department of Biomedical Technology, Faculty of Biomedical Engineering, Czech Technical University in Prague, Sítná Squere 3105, Kladno, Czech Republic, +420739318121; 2 Faculty of Health Studies, Technical University of Liberec, Liberec, Czech Republic

**Keywords:** electronic health records, interoperability, health information exchange, qualitative research, stakeholder participation, European Health Data Space, Integrating the Healthcare Enterprise Cross-Enterprise Document Sharing, IHE XDS, Czech Republic

## Abstract

**Background:**

Operational health data interoperability in post-transition health systems requires not only technical standards but also enforceable governance arrangements. Affinity domains, as defined in the IHE XDS (Integrating the Healthcare Enterprise Cross-Enterprise Document Sharing) framework, represent a structured organizational-technical model for cross-enterprise document sharing. However, evidence from Central and Eastern Europe on their governance feasibility and implementation readiness remains limited, particularly in systems characterized by institutional fragmentation and evolving regulatory mandates.

**Objective:**

This study aimed to examine stakeholder perceptions of the prerequisites, risks, benefits, and governance conditions for implementing affinity domains in the Czech health care system. The study further sought to identify system-level readiness factors relevant to national interoperability initiatives in the context of the European Health Data Space regulation.

**Methods:**

We conducted 18 semistructured interviews between January 2025 and April 2025 with policymakers, regional health authorities, health care providers, health insurance funds, health IT vendors, and independent experts. Participants were selected using purposive and snowball sampling to ensure institutional diversity. Interviews explored governance roles, legal accountability for shared data, enforcement of standards, financing models, and technical readiness. Data were analyzed inductively using thematic analysis in MAXQDA 24 (VERBI Software GmbH), supported by dual coding, consensus discussions, and reflexive memoing. Inductively derived themes were subsequently interpreted against core governance dimensions of affinity domains (rule-making authority, membership and participation, accountability, enforcement, and trust).

**Results:**

Five major thematic categories emerged: (1) ambiguous roles and responsibilities marked by fragmented mandates and vendor influence; (2) perceived risks, including institutional distrust, legal uncertainty regarding liability, technical fragmentation, and vendor lock-in; (3) system-level prerequisites such as the need for an empowered coordinating authority, binding interoperability standards (IHE XDS and HL7 FHIR [Health Level 7 Fast Healthcare Interoperability Resources]), sustainable financing, and human resource capacity; (4) perceived benefits, including improved continuity of care, reduced duplication, and enhanced transparency; and (5) structural implementation barriers including political discontinuity, weak enforcement mechanisms, and limited stakeholder engagement. Across stakeholder groups, feasibility was consistently linked to 3 actionable readiness conditions: clearly assigned responsibility and liability for shared data, enforceable technical requirements supported by certification or audit mechanisms rather than voluntary compliance, and financing models addressing both IT infrastructure and organizational change.

**Conclusions:**

Affinity domains are recognized as a viable interoperability model in the Czech Republic, but their implementation is constrained primarily by governance deficits rather than technical immaturity. Establishing an empowered coordinating authority, piloting enforceable domain structures, and aligning national governance with European Health Data Space requirements represent necessary steps toward operational interoperability. These findings provide empirically grounded insights into the governance conditions required for scalable health information exchange in post-transition health systems.

## Introduction

The digital transformation of health care systems is widely recognized as a strategic priority across Europe, aiming to improve care quality, efficiency, and patient safety while enabling sustainable system governance [1]. A key enabler of this transformation is the establishment of interoperability infrastructure that allows secure and standardized exchange of medical information among health care providers, patients, and administrative entities [2]. Within this context, the concept of affinity domains, as defined within the IHE XDS (Integrating the Healthcare Enterprise Cross-Enterprise Document Sharing) architecture, constitutes a sociotechnical construct that combines a technical framework for cross-enterprise document sharing with an explicit governance arrangement. Beyond interoperability per se, affinity domains are characterized by shared rule-making, defined membership, accountability for data stewardship, and enforcement mechanisms governing participation and compliance among involved organizations [3,4]. While health information exchange (HIE) or electronic health record (EHR) interoperability primarily refers to the technical ability of systems to exchange data, an affinity domain additionally defines shared governance rules, membership criteria, accountability, and enforcement mechanisms for participating organizations. In this paper, we use the term “interoperability infrastructure” to refer broadly to the technical and governance ecosystem enabling cross-enterprise data exchange, “HIE” to denote operational exchange systems, and “affinity domains” to describe a governance-anchored interoperability model as conceptualized in the IHE XDS framework.

While the implementation of affinity domains has been successfully institutionalized in several Western and Northern European countries—such as Austria (Elektronische Gesundheitsakte [ELGA]), Switzerland (Elektronisches Patientendossier [EPD]), the Netherlands (Landelijk Schakelpunt), and Sweden (Národní plán obnovy)—their adoption across Central and Eastern Europe remains limited and fragmented. Although conceptual frameworks and technical standards such as IHE XDS have been available for over a decade [4], countries in this region continue to face systemic, organizational, and legislative barriers to real-world deployment [5]. The Czech Republic, despite recent improvements in its eHealth strategy and digital infrastructure, continues to lack the operational governance and institutional capacity required to implement affinity domains at scale. In practical terms, the Czech Republic currently lacks a national HIE system comparable to ELGA or EPD, and interoperability initiatives remain primarily project-based or regionally bounded. Although Act No. 325/2021 Collection of Laws established a legal framework for digital health governance, implementation mechanisms, certification procedures, and clearly mandated coordinating authorities remain only partially operational. As a result, interoperability is shaped more by institutional negotiation and vendor configurations than by enforceable national governance structures.

Since the early 2000s, the Czech health care system has initiated multiple digitalization efforts—most prominently the Internet Health Information Portal (Internetový zdravotní informační portál [IZIP]) and ZDRAVeL platforms—but these have largely failed to achieve widespread adoption due to fragmented governance, insufficient standardization, institutional resistance, and lack of sustained stakeholder engagement. The IZIP system, launched in 2004, was a pioneering national web-based EHR that demonstrated clear potential but achieved limited integration and impact in routine care [6]. Its successor, ZDRAVeL, introduced improved functionality but similarly failed to secure institutional alignment and embed itself into clinical workflows [7]. At present, the only digital health applications with nationwide coverage and consistent daily use are the Outpatient Electronic Prescription System [8] and the Digital Imaging and Communications in Medicine (DICOM) image exchange platform [9]. Other systems exist but remain fragmented, region-specific, or limited to pilot projects, which restrict large-scale interoperability. A notable example of a successful local secure health data exchange platform is the eMeDOcS (Exchange Medical Documents System) project [10]. The eMeDOcS system is an electronic platform designed to enable structured data exchange across affinity domains, supporting interoperability and standardized clinical communication. It is mainly used by hospitals in the Vysočina Region. The eMeDOcS system is based on the ICZ ISAC (Integration, Share and Communication System) platform [11], which enables easy integration between EHRs and other specific systems, including telemedicine solutions [12].

More recent evaluations note that although the COVID-19 pandemic accelerated telemedicine adoption, this progress remains fragmented and contingent, hampered by legal uncertainty and the absence of a functional interoperability infrastructure [13]. Comparable challenges have been documented in other post-transition systems, where weak enforcement mechanisms, unclear institutional mandates, and limited technical standardization obstruct effective interoperability [14,15]

Despite these conceptual and technical advances, affinity domains have not progressed beyond policy discussions in the Czech Republic, and no operational implementation currently exists. Several domain experts note that while the IHE-affinity domain concept is acknowledged and debated at the policy level in the Czech Republic, actual system-level implementations are deferred due to government ambivalence about central steering versus federated emergence, and the absence of pilot registries or unified patient identity mechanisms. Similarly, an interoperability pilot (Semantic Interoperability Platform) launched in the mid-2000s demonstrated the technical readiness to adopt international standards but failed to scale due to institutional fragmentation and a lack of sustained governance frameworks [16].

As of 2024, electronic medical record systems are widely adopted at the institutional level in the Czech Republic, with nearly all hospitals and most outpatient providers operating some form of digital documentation. However, these systems remain predominantly vendor-specific and lack cross-enterprise interoperability. No nationwide HIE system comparable to ELGA or EPD is operational. Regional exchange initiatives, such as eMeDOcS in the Vysočina Region, demonstrate technical feasibility using IHE XDS–based architectures, but their implementation remains geographically limited and institutionally fragmented. National digital infrastructure is currently centered around selected vertical services (ePrescription and DICOM image exchange), while unified patient identity management, master patient indexing, and certification mechanisms for interoperability remain underdeveloped. Although the legal framework for digital health governance was established by Act No. 325/2021 Collection of Laws, operational governance structures and mandatory interoperability enforcement are still evolving.

Despite the availability of technical standards and formal policy documents, existing literature [17] provides limited insight into how the governance attributes of affinity domains, such as rule-making authority, accountability, participation, enforcement, and trust, are interpreted and operationalized by institutional actors in practice. While affinity domains are technically defined within the IHE XDS framework, these governance dimensions are only partially reflected in legal texts or technical specifications and tend to emerge through organizational interactions, negotiation processes, and implementation experiences. Existing research has primarily focused on legal or technical aspects rather than governance practices. To address this gap, we adopted a qualitative design based on stakeholder interviews, which enables analysis of how abstract interoperability models translate into real-world decision-making contexts and how governance responsibilities are perceived and enacted by key system actors [18].

Therefore, in this study, we aim to contribute to the evolving discourse on digital health governance in post-transition health care systems by (1) presenting key findings from stakeholder perceptions regarding the implementation of affinity domains in Czech health care, (2) analyzing these findings in comparison with selected European experiences, and (3) offering evidence-informed recommendations for advancing interoperability through affinity domains as part of the national eHealth policy.

## Methods

### Study Design and Objective

This study used a qualitative, exploratory design using semistructured interviews and stakeholder analysis to examine perceptions, expectations, and barriers related to the implementation of affinity domains in the Czech health care system. The qualitative approach was selected to capture governance-related attributes of affinity domains—such as the allocation of roles, accountability for shared data, enforcement mechanisms, and trust relationships—that are central to their feasibility but are not directly observable through policy documents or technical specifications. The research objective was to identify enabling and constraining factors from the perspective of key stakeholder groups and derive actionable insights to inform policy and strategy. At the start of each interview, participants were provided with a standardized, brief explanation of an “affinity domain” based on IHE XDS, emphasizing both its governance components (shared policies, membership, accountability, and enforcement) and its technical scope (cross-enterprise document sharing under agreed profiles). Participants were asked to reflect on prerequisites, risks, benefits, and barriers specifically in relation to this defined model. Where participants referred to interoperability challenges more broadly, such statements were retained in the analysis only insofar as they directly constrained or enabled the feasibility of implementing affinity domains in practice. The study adhered to the COREQ (Consolidated Criteria for Reporting Qualitative Research) 32-item checklist to ensure methodological transparency (Checklist 1) [19].

### Research Team and Reflexivity

Interviews were conducted by PH and IF, both of whom have formal training in qualitative health research and prior experience conducting semistructured interviews in digital health policy projects. JB co-led the analytic phase. A brief relationship with some participants existed through professional networks used for initial identification; however, no direct supervisory or contractual dependency was present. Participants were informed about the researchers’ institutional affiliation, study aims, and interest in interoperability governance. To mitigate potential bias, we used an a priori protocol, a piloted interview guide, maintained an audit trail, and implemented dual independent coding with consensus resolution; reflexive memos documented assumptions and analytic decisions throughout. We did not compute formal intercoder reliability coefficients, opting instead for iterative consensus and peer debriefing.

### Stakeholder Framework and Selection

The selection of stakeholder groups was based on a combined analytical and policy-relevant approach, aligned with established digital health governance frameworks, including multilevel governance approaches and actor-based interoperability models [17,18]. Stakeholders were defined as individuals or institutions that directly influence or are affected by the implementation of national eHealth infrastructure, specifically interoperability components such as affinity domains. Stakeholder groups were selected to reflect core governance roles within an affinity domain, including regulatory authority, payers, providers, vendors, and independent technical experts. These roles correspond to the key governance dimensions of affinity domains, namely rule-setting authority (Ministry of Health [MoH]), implementation coordination (regional health authority [RHA]), operational participation (health care providers), financing and regulatory oversight (health insurance fund [HIF]), technical standard implementation (health IT vendors), and advisory or normative alignment (independent consultants and expert advisors). Inclusion criteria were (1) active involvement in drafting or implementing national or regional eHealth strategies, (2) responsibility for management or governance of hospital information system or interoperability solutions, or (3) technical or advisory roles in pilots or standard-setting activities. Exclusion criteria included the absence of formal responsibility for digital health governance or purely end-user roles without decision authority. Stakeholders were identified through a purposive sampling strategy supplemented by snowball sampling [20]. First, 6 stakeholder categories were defined a priori based on their formal roles in national and regional digital health governance. Within these predefined categories, individuals were primarily identified through purposive selection based on their formal responsibilities, documented involvement in national or regional eHealth initiatives, and participation in legislative or technical working groups. Snowball sampling was subsequently used as a supplementary strategy to identify additional relevant informants, particularly within the independent expert group and in cases where formal role-based identification was insufficient. The recruitment process continued until adequate coverage of all predefined categories was achieved. This resulted in a final pool of 25 identified candidates, of whom 22 were formally invited; 4 declined due to time constraints, resulting in 18 completed interviews.

To ensure both diversity of perspectives and relevance to the Czech health care context, the following 6 stakeholder groups were identified.

Table 1 shows the stakeholder groups and corresponding inclusion criteria applied in a qualitative semistructured interview study examining the implementation of IHE XDS–based affinity domains in the Czech health care system. Eighteen interviews were conducted between January 2025 and April 2025. Participants were recruited using purposive and snowball sampling to represent regulatory, financing, service provision, health IT vendor, and advisory roles. Detailed participant characteristics are provided in Multimedia Appendix 1.

**Table 1.** Stakeholder selection criteria (N=18).

Stakeholder group	Role in eHealth system	Inclusion criteria	Participants, n (%)
Ministry of Health^a^, Czech Republic	Policy development, legislation, and national coordination	Direct involvement in drafting or implementing Act No. 325/2021 Collection of Laws or national eHealth strategies	3 (16.6)
RHAs^h^	Regional eHealth implementation and funding oversight	Responsibility for managing or co-funding regional eHealth projects, or an active role in local Hospital Information System governance	3 (16.6)
Health care providers	Operational users of interoperability infrastructure	Senior managers or digital transformation leads in hospitals participating in national eHealth projects or data-sharing pilots	4 (22)
HIF^c^	Data consumers, payers, and system regulators	Participation in interoperability-related initiatives; responsibility for data security or claims data infrastructure	2 (11)
Health IT vendors	System developers and integrators of interoperability solutions	Experience with IHE^d^ XDS^e^ or HL7^f^ FHIR^g^ implementations; vendors of Hospital Information Systems used in the Czech or European Union interoperability frameworks	4 (22)
Independent consultants and expert advisors	Strategic or technical advising, legislation support	Recent collaboration with MoH or regions on legislative drafting, standard setting, or coordination of digital health pilot programs	2 (11)

a MoH: Ministry of Health.

b RHA: regional health authority.

c HIF: health insurance fund.

d IHE: Integrating the Healthcare Enterprise.

e XDS: Cross-Enterprise Document Sharing.

f HL7: Health Level 7.

g FHIR: Fast Healthcare Interoperability Resources (HL7).

The purposive strategy targeted participants with substantial experience or formal responsibility in health information systems, eHealth projects, or regulatory implementation. Recruitment prioritized individuals actively engaged in national-level working groups, projects funded through the Integrated Regional Operational Program or National Recovery Plan (Národní plán obnovy) schemes, and the development of Czech national digital health legislation. This ensured that all selected informants had first-hand knowledge of operational, regulatory, or strategic aspects of digital health implementation.

As noted above, snowball sampling complemented the primary purposive strategy by enabling the inclusion of additional consultants and advisors involved in recent pilot initiatives or standard-setting activities. Recruitment followed a structured, multiphase logic: initial identification through professional networks, follow-up invitations by email, and confirmation of eligibility against the inclusion criteria.

The final sample reflected both policymaking and operational perspectives, as well as representation from public and private sector stakeholders. While not statistically representative, the composition was sufficient to achieve thematic saturation across the predefined analytical categories, thereby increasing the credibility and contextual depth of the findings. Group-level distributions (MoH, RHAs, health care providers, HIFs, health IT vendors, and independent consultants and expert advisors) are detailed in Checklist 1 to support COREQ-aligned reporting.

### Data Collection

Data were collected through semistructured interviews, providing a flexible yet systematic approach to exploring stakeholder perspectives. Interviews were conducted between January 2025 and April 2025. An interview guide was developed based on a review of relevant literature and pilot-tested with 2 external experts, which led to minor refinements in question wording and flow. Interviews were scheduled for approximately 50 minutes and were conducted online via secure video conferencing tools to accommodate participants’ geographical distribution.

All interviews were audio-recorded with participants’ consent, and detailed field notes were authored immediately after each session. No repeat interviews were conducted. Only the interviewer and participant were present during each interview; no third parties attended. Interview transcripts were returned to all participants for member checking. Of the 18 participants, 12 provided feedback or explicit confirmation, and minor clarifications were incorporated where relevant. The remaining participants did not request modifications. The full semistructured interview guide, including main questions and illustrative probes, is provided in Multimedia Appendix 2 to enhance transparency regarding question framing and potential prompting effects.

The interview guide focused on 4 main domains: stakeholders’ perceptions of opportunities and barriers in digital health implementation, experiences with project governance and interinstitutional collaboration, anticipated impacts of national digital health strategies and legislation, and suggestions for improving effectiveness, efficiency, and trust in digital health projects.

Data saturation was monitored throughout the process and was judged to have been achieved after the 12th interview; subsequent interviews confirmed existing themes without generating substantial new insights. A saturation grid documenting the last occurrence of novel codes is included in Multimedia Appendix 3. A summary of preliminary themes was also shared with participants, who provided confirmation or minor clarifications.

### Data Analysis

To support construct validity, the concept of affinity domains was analytically specified through a set of governance and technical dimensions derived from the IHE XDS framework and relevant digital health governance literature [3,4,17].

Table 2 shows the conceptual specification of governance and technical dimensions constitutive of affinity domains, which were used as an interpretive reference in this qualitative interview study conducted within the Czech health care system (18 interviews; January 2025-April 2025). The dimensions were derived from the IHE XDS framework and relevant digital health governance literature, serving as an analytical lens rather than a deductive coding framework.

**Table 2.** Conceptual specification of affinity domain dimensions used as an interpretive reference.

Affinity domain dimension	Conceptual basis	Relevance for analysis
Shared rule-making	IHE^a^ XDS^b^ governance model; digital health governance literature	Framing how rules and standards are defined and legitimized
Membership and participation	Affinity domain concept; institutional governance	Understanding inclusion or exclusion of actors
Accountability and liability	Governance and health law literature	Interpreting responsibility for shared data
Enforcement mechanisms	EHDS^c^ and interoperability governance debates	Assessing compliance beyond voluntary adoption
Trust relationships	Trust and legitimacy literature in digital health	Explaining willingness to participate
Technical scope (IHE XDS)	IHE XDS architecture	Anchoring findings in document-based exchange

a IHE: Integrating the Healthcare Enterprise.

b XDS: Cross-Enterprise Document Sharing.

c EHDS: European Health Data Space.

These dimensions were not applied as a deductive coding scheme but served as an interpretive reference for examining how inductively derived themes relate to the core attributes of affinity domains. This approach preserved the inductive nature of the thematic analysis while ensuring conceptual alignment with the affinity domain construct.

Interview transcripts were subjected to a thematic analysis using MAXQDA 24 (VERBI Software GmbH, Berlin, Germany, version 24.1.0) to support the organization and coding of qualitative data. An initial inductive codebook was developed and iteratively refined; the final coding tree with major themes and subthemes is provided in Multimedia Appendix 4. Minor and divergent views were also reported, ensuring that less frequent but important perspectives were not overlooked. A worked example (quote → in-vivo code → category → theme) is provided in Multimedia Appendix 5, along with a table of representative quotes by theme and subtheme. The higher-order thematic categories were subsequently interpreted in relation to the governance dimensions constitutive of affinity domains.

The analysis followed a multistep, reflexive process consistent with best practices in qualitative health research:

- Familiarization: researchers conducted repeated readings of transcripts and field notes to identify initial patterns.
- Coding: inductive, in vivo codes were applied independently by 2 coders (PH and IF); discrepancies were discussed until consensus was reached, with an audit trail documenting decisions. We did not compute a formal intercoder reliability coefficient; instead, we used iterative consensus meetings and peer debriefing to ensure dependability.
- Theme development: codes were clustered into broader thematic categories capturing recurrent stakeholder perspectives. Some empirical phenomena spanned multiple domains and were analytically differentiated according to their primary governance implications. To enhance readability in the “Results” section, conceptually related phenomena are discussed under their primary governance function, with cross-references where appropriate. For instance, vendor dominance was interpreted under “roles and responsibilities” as an issue of rule-making authority, whereas vendor lock-in was classified under “perceived risks” as a structural constraint affecting enforceability and participation. This approach ensured conceptual clarity while maintaining alignment with governance attributes central to affinity domains.
- Verification: a third qualitative expert reviewed the coding scheme and thematic structure to ensure rigor and validity. Conceptually related codes (eg, vendor dominance and vendor lock-in, and legal uncertainty) were critically examined during iterative refinement. Where retained in separate higher-order categories, distinctions were based on their primary governance function (eg, structural power asymmetry vs enforceability constraints), thereby avoiding post hoc theme allocation. The use of MAXQDA facilitated systematic data management and the creation of thematic maps. The relative prominence of each domain was visualized through frequency-weighted displays, supporting the interpretation and presentation of the findings.

Quotations in the “Results” section are labeled using a dual system: a unique respondent code (R1-R18) and the corresponding stakeholder group (MoH, RHAs, health care providers, HIFs, health IT vendors, and independent consultants and expert advisors). Each code was assigned to a single participant and used consistently throughout the analysis. The distribution of respondent codes mirrors the sample composition described in Table 1: MoH (n=3), RHAs (n=3), health care providers (n=4), HIFs (n=2), health IT vendors (n=4), and independent consultants and expert advisors (n=2). This labeling strategy enhances the transparency of reporting while preserving participant anonymity. Where appropriate, we report countervailing or minority views to avoid over-generalization.

To contextualize the Czech findings, we conducted a focused desk-based review of selected European countries with operational HIE systems comparable to IHE XDS–based affinity domains (Austria, Switzerland, the Netherlands, Sweden, and Belgium). Countries were selected purposefully to reflect different governance architectures (centralized, federated, and regional) and based on the availability of official documentation and peer-reviewed analyses.

Data were extracted from national eHealth portals, legislative documents, and relevant academic literature. Information was synthesized descriptively, focusing on governance structures, legal mandates, implementation scopes, and coordination mechanisms. This component served as a contextual comparison and did not constitute a separate systematic review.

### Ethical Considerations

The study was approved by the ethics committee of the Faculty of Biomedical Engineering under protocol C59/2025. Research procedures were conducted in compliance with local legislation and institutional requirements [21]. The ethical approval covered the recruitment of participants from external public and private institutions in their professional capacity. No additional institutional approvals were required beyond individual informed consent.

All participants received an information sheet explaining the study’s purpose, procedures, and their rights, including confidentiality and the voluntary nature of participation. Written informed consent was obtained before data collection. Participant identities were anonymized and institutional affiliations were masked to protect confidentiality and comply with data protection standards. Audio files and transcripts were stored on secure institutional servers with restricted access, and deidentified transcripts were used for analysis. There was no compensation awarded.

## Results

### Overview

The thematic analysis yielded five major categories concerning the implementation of affinity domains in the Czech health care system: (1) roles and responsibilities, (2) perceived risks, (3) system-level prerequisites, (4) perceived benefits and opportunities, and (5) implementation barriers. These categories capture institutional expectations, governance concerns, and perceived feasibility conditions articulated across stakeholder groups.

The categories are presented thematically according to the dominant governance implications identified across stakeholder groups. Where phenomena intersected multiple governance dimensions (eg, vendor influence or legal uncertainty), they are discussed under their primary governance function.

**Figure 1.**
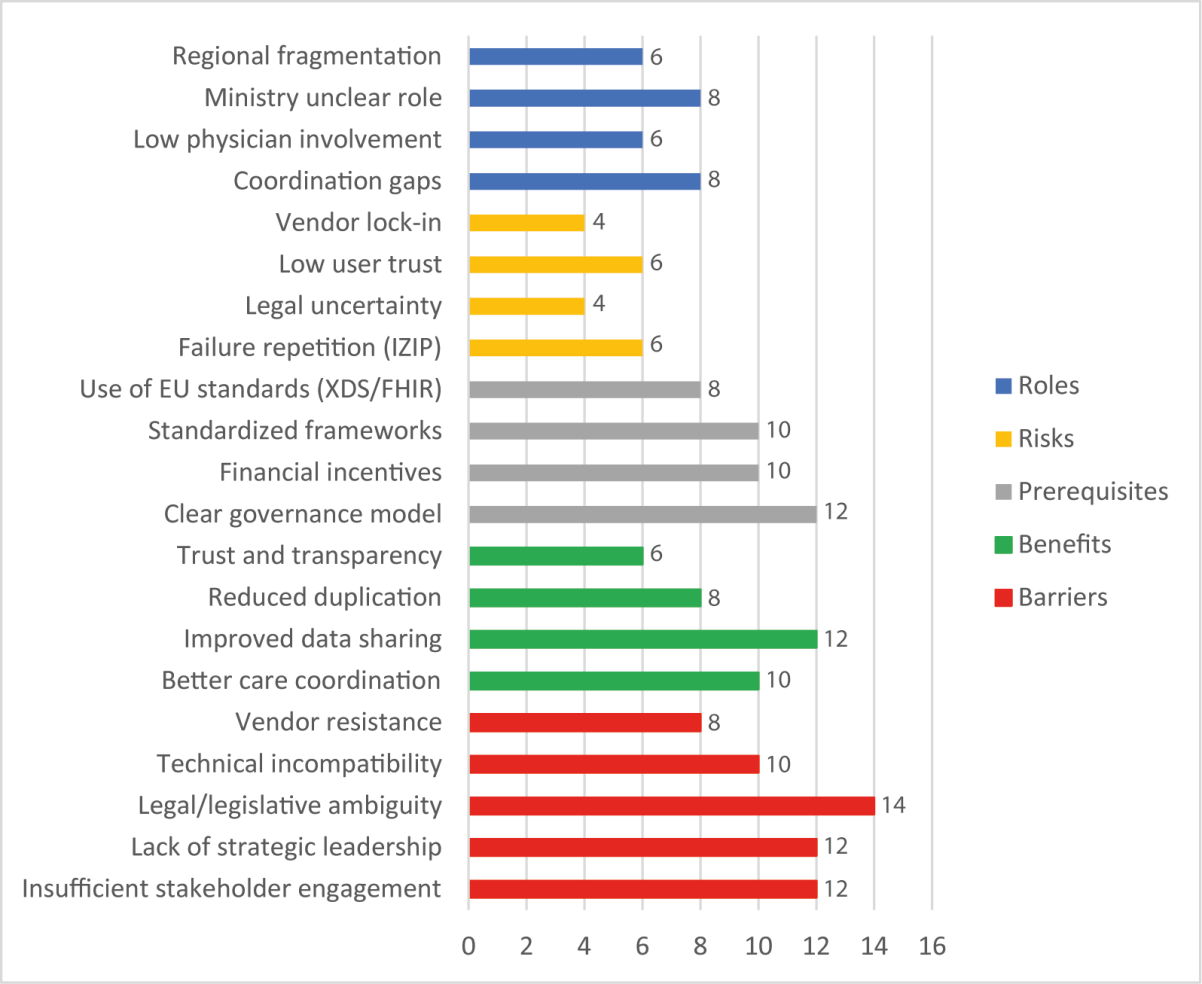
Distribution of major thematic categories derived from stakeholder interviews (N=18 interviews). FHIR: Fast Healthcare Interoperability Resources; HL7: Health Level 7; IZIP: Internetový zdravotní informační portál; MoH: Ministry of Health; XDS: Cross-Enterprise Document Sharing.

Figure 1 illustrates the distribution of major thematic categories derived from 18 semistructured stakeholder interviews conducted within the Czech health care system between January and April 2025. The bars represent the frequency of coded text segments assigned to each theme across the dataset. The values reflect relative thematic prominence and do not indicate the number of participants endorsing a theme or statistical significance.

### Thematic Categories

#### Roles and Responsibilities

The analysis of stakeholder interviews revealed a systemic ambiguity in the definition and operationalization of roles within the governance of affinity domains in the Czech health care system. While the MoH formally holds responsibility for strategic direction in digital health, respondents consistently emphasized its limited engagement in operational implementation. As 1 participant noted:

*The Ministry sets strategies but does not enforce them. We’re left guessing what they really want.*

[R18, independent consultant and expert advisor]

Several participants highlighted the absence of an empowered intermediary body with the technical and legal authority to coordinate interoperability initiatives—a role that, in other countries, is fulfilled by institutions such as Gematik (Germany).

At the regional level, fragmentation emerged as a critical barrier. Regional IT departments reportedly operate with minimal alignment, resulting in uncoordinated and heterogeneous solutions. This decentralization exacerbates interoperability issues and complicates accountability. According to another respondent:

*Each region develops its own system, with no national integration in sight. We’re building isolated islands.*

[R4, RHA]

The lack of nationally endorsed technical and procedural standards reinforces these inconsistencies.

Stakeholders also identified a pronounced disconnect between digital governance structures and clinical end-users. Physicians, despite being primary users of EHRs, are reportedly excluded from planning and decision-making processes, as 1 respondent explained:

*We receive new systems and tools, but no one asks whether they match how we work. That’s why so many doctors ignore them.*

[R7, health care provider]

This exclusion leads to suboptimal system design and limited adoption.

Finally, vendor dominance was perceived as a significant factor undermining coherent governance. In many facilities, technology suppliers exert considerable influence, effectively establishing de facto standards and resisting external integration. As 1 respondent put it:

*In some hospitals, the vendor decides more than the IT department. They lock the system and then dictate what’s possible.*

[R13, health IT vendor]

The absence of regulatory mechanisms to counterbalance these dynamics contributes to role diffusion and undermines long-term governance sustainability. This asymmetry also underpins later concerns regarding vendor lock-in and resistance during implementation.

In governance terms, this ambiguity signals the absence of clearly defined rule-making authority, membership criteria, accountability allocation, and enforceable coordination mechanisms that are constitutive of affinity domains as structured interoperability arrangements.

#### Perce ived Risks

Interviewees articulated a range of interrelated risks that threaten the feasibility and sustainability of affinity domains. The most frequently cited concern was reputational damage and institutional distrust rooted in the failed IZIP initiative. As 1 respondent recalled:

*IZIP left a scar. We’ve all seen what happens when things go wrong—and no one wants to be blamed again.*

[R17, independent consultant and expert advisor]

Respondents noted that the legacy of IZIP continues to generate reluctance among stakeholders to participate in digital health projects, particularly those lacking transparent governance and legal protection.

Legal uncertainty regarding liability for shared data was another dominant theme. Many stakeholders expressed concern over the absence of clear accountability frameworks for data integrity and clinical liability once information is shared across domains. As 1 respondent emphasized:

*If a patient is harmed due to outdated or missing data, who will take responsibility? No one wants to be exposed.*

[R8, health care provider]

This regulatory vacuum exacerbates institutional risk aversion, particularly in environments where errors could directly affect patient safety.

Technical risks were linked to the heterogeneous landscape of health IT systems and the lack of mandatory, enforceable interoperability standards. Interviewees reported variability in the implementation of HL7 FHIR and IHE profiles, often contingent upon vendor willingness rather than policy mandates. As 1 respondent observed:

*Everyone does their own thing. Some use FHIR; some don’t. There’s no technical backbone that guarantees compatibility.*

[R14, health IT vendor]

The absence of a national HIE system or compliance framework further limits reliable data exchange.

Building on earlier concerns about vendor dominance in rule-setting processes, stakeholders described vendor lock-in as a structural risk limiting enforceable participation. One respondent remarked, “Why would a vendor enable data sharing if it means losing business to a competitor?” (R15, health IT vendor). Additionally, low trust among clinical professionals—linked to past failures and limited inclusion in governance—was reported as a persistent obstacle to stakeholder engagement. As 1 respondent stated:

*It feels like another bureaucratic project we’re supposed to accept without discussion. That erodes trust, not builds it.*

[R9, HP]

These risks indicate fragile institutional preconditions for affinity domains, particularly with respect to trust, liability clarity, and enforceable participation rules.

#### System-Level Prerequisites

Participants outlined several prerequisites critical for the successful implementation of affinity domains. Chief among these was the establishment of a functionally independent governance structure capable of coordinating the technical, legal, and strategic aspects of interoperability deployment. Respondents advocated for a body with a defined mandate, cross-sectoral legitimacy, and operational capacity. As 1 respondent pointed out:

*You can’t just publish a strategy and hope that hospitals will fall in line. Someone has to own it, drive it, and manage it.*

[R2, MoH]

Funding was highlighted as a key enabling factor, given the substantial costs of system upgrades, training, and organizational redesign. According to 1 respondent:

*We’re not against interoperability. But who’s going to pay for all the technical and organizational changes it requires?*

[R11, HIF]

Incentive structures, including European Union–linked funding or performance-based reimbursement, were proposed as potential levers to foster participation.

Technical prerequisites included the uniform adoption of recognized standards such as HL7 FHIR and IHE XDS, as well as the creation of centralized compliance frameworks, reference implementations, and certification schemes. Interviewees also emphasized the need for inclusive interoperability beyond hospitals, incorporating social services, emergency care, and public health actors. As 1 respondent highlighted:

*You can’t build a regional domain if the social care system is left out. It’s half of the equation.*

[R5, RHA]

Such integration would necessitate coordinated policymaking across ministries.

Human resource capacity was also noted as a critical constraint. Respondents cited a shortage of professionals with the requisite skills to implement and manage interoperability solutions at both the institutional and regional levels. As 1 respondent explained:

*The biggest bottleneck isn’t technology. It’s people who understand the standards and can actually implement them.*

[R18, independent consultant and expert advisor]

These prerequisites correspond to the foundational governance conditions of affinity domains, including the need for an empowered coordinating authority, formally recognized participation rules, technical compliance enforcement, and sustainable operational capacity.

#### Perceived Benefits and Opportunities

The perceived benefits of affinity domains were principally associated with improved clinical coordination, operational efficiency, and patient safety. The most prominent advantage cited by stakeholders was seamless access to structured patient data across institutional boundaries. Respondents described scenarios in which inadequate information sharing resulted in redundant diagnostics, delayed care, or clinical uncertainty. As 1 respondent described:

*If a patient comes from another hospital, I usually start from scratch. Even allergy history is often missing.*

[R10, health care provider]

Affinity domains were also perceived as a mechanism to reduce redundant diagnostic procedures, particularly in oncology and chronic care, where patients often traverse multiple providers. One respondent reported:

*We see the same CT scans being done twice just because the systems don’t talk. That’s both costly and unethical.*

[R8, health care provider]

In addition to financial savings, stakeholders emphasized the ethical imperative to avoid unnecessary patient burdens.

Improved continuity of care and support for integrated service delivery was also recognized. Interoperable access to discharge summaries, medication histories, and treatment plans was seen as essential for primary care physicians managing complex patients. As 1 respondent mentioned, “A GP who sees a patient after hospital discharge should not have to guess what happened during hospitalization” (R7, health care provider).

Some respondents identified the potential for interoperability to facilitate system-level accountability through audit trails and standardized documentation. According to 1 respondent:

*If we can see who accessed what, when, and why, it becomes easier to ensure compliance and trace responsibility.*

[R1, MoH]

Secondary uses of data, including research, surveillance, and planning, were identified as long-term opportunities, albeit contingent on the establishment of trust and robust legal and technical safeguards. As 1 respondent suggested:

*Affinity domains could eventually feed anonymised data into registries or support AI models. But that’s the next step.*

[R17, ICEA]

These anticipated benefits reflect the governance potential of affinity domains to structure accountability, enable traceable exchanges, and foster coordinated cross-institutional participation.

#### Implementation Barriers

Stakeholders identified multiple barriers hindering the adoption of affinity domains, spanning legal, organizational, technical, and cultural domains. The lack of a comprehensive legal framework was cited as the most significant impediment. Beyond the liability-related uncertainty described under the “Perceived Risks” section, respondents also identified broader legal uncertainty—particularly regarding consent procedures and data stewardship—as an operational barrier. As 1 respondent stated:

*We operate in legal gray zones. Everyone is afraid of making a mistake, so they’d rather do nothing.*

[R6, RHA]

Political instability and weak leadership continuity were also seen as undermining implementation. Respondents described repeated strategic resets with governmental turnover, leading to policy fatigue and limited stakeholder confidence. One respondent observed:

*There’s no continuity. One government introduces a concept, the next one cancels it. That’s how we fail by design.*

[R18, independent consultant and expert advisor]

Technical fragmentation, particularly the coexistence of proprietary, noninteroperable systems, was noted across both hospitals and outpatient facilities. The absence of national technical infrastructure (eg, a HIE system) was seen as a structural constraint on scalability. As 1 respondent described:

*Every vendor builds their own castle. And no one checks if the drawbridges align.*

[R16, health IT vendor]

In operational terms, stakeholders also described vendor resistance as a practical barrier during implementation, including commercial disincentives such as excessive application programming interface fees and limited technical transparency. As 1 respondent explained:

*If interoperability isn’t part of the contract, don’t expect vendors to care.*

[R15, HV]

Finally, limited stakeholder engagement—especially among frontline providers—was identified as a barrier to implementation. The top-down nature of digital health policy development was seen as excluding key actors, undermining system ownership, and contributing to resistance at the operational level. According to 1 respondent:

*Doctors, nurses, even IT departments—most of them hear about new projects for the first time at the rollout stage*. [R9, health care provider]

Taken together, these barriers reflect a structural misalignment between current institutional arrangements and the governance logic required for affinity domains, particularly in terms of enforceability, defined domain membership, leadership continuity, and coordinated policy execution.

## Discussion

### Summary of Main Findings

Our findings indicate that the feasibility of affinity domains in the Czech health care system is primarily shaped by governance-related constraints rather than by the absence of technical standards. Across stakeholder groups, barriers clustered around unclear institutional mandates, weak enforcement mechanisms, fragmented implementation structures, and limited stakeholder inclusion—factors that collectively undermine trust and coordination required for cross-enterprise data sharing.

### Synthesis of Themes and Policy Implications

To enhance the policy relevance and readability of the findings, we synthesized the 5 empirical themes into a structured overview linking governance gaps to corresponding policy implications. While the themes emerged inductively from stakeholder interviews, their interpretation reveals actionable dimensions relevant to affinity domain implementation.

Table 3 shows the synthesis of empirically derived thematic categories and their corresponding governance implications for affinity domain implementation in the Czech health care system. The table is based on findings from 18 semistructured stakeholder interviews conducted between January 2025 and April 2025 and reflects author-derived implications grounded in qualitative analysis rather than direct participant recommendations.

**Table 3.** From thematic findings to policy implications for affinity domain implementation.

Empirical theme	Governance gap identified	Policy implication
Roles and responsibilities	Ambiguous mandates Absence of empowered coordinating body Vendor dominance	Establish an independent national interoperability authority with clear rule-making and enforcement powers
Perceived risks	Legal uncertainty regarding liability Institutional distrust Vendor lock-in	Clarify liability frameworks Introduce binding interoperability requirements Create contractual enforcement mechanisms
System-level prerequisites	Lack of certification frameworks Inconsistent standard adoption Limited human capacity	Implement mandatory technical standards (IHE^a^ XDS^b^ and HL7^c^ FHIR^d^) supported by certification, audit, and national reference implementations
Perceived benefits	Recognized value for continuity of care and accountability, but unrealized due to governance gaps	Use pilot affinity domains to demonstrate measurable clinical and operational benefits
Implementation barriers	Political discontinuity Fragmented regional approaches Weak stakeholder engagement	Introduce phased pilots with co-design mechanisms and multilevel governance coordination aligned with EHDS^e^.

a IHE: Integrating the Healthcare Enterprise.

b XDS: Cross-Enterprise Document Sharing.

c HL7: Health Level 7.

d FHIR: Fast Healthcare Interoperability Resources.

e EHDS: European Health Data Space.

### Comparison With International Literature

The study identifies several systemic, structural, and contextual obstacles that complicate the implementation of affinity domains within the Czech health care system. While some of these challenges correspond to broader trends observed across European countries, the Czech case is distinguished by shortcomings in governance architecture, legal frameworks, and stakeholder involvement.

Although affinity domains are not always explicitly addressed in major European policy and governance studies, the structural conditions underpinning their creation and sustainability are frequently analyzed. Affinity domains, as defined by the IHE XDS framework, require not only interoperable technical architectures but also clearly defined governance structures, implementation standards, and legal-operational certainty. Recent literature in digital health governance thus offers critical insights into the enabling—or inhibiting—factors for their deployment.

To contextualize the Czech findings, we purposively selected European countries representing distinct governance architectures of affinity domain–like models (centralized, federated, semicentralized, and regional). These cases were chosen to reflect variation in institutional authority, legal mandates, and implementation maturity, thereby enabling analytical comparison with the governance gaps identified in the Czech interviews.

Table 4 shows a comparative overview of affinity domain–like interoperability governance models in selected European countries, based on desk-based document review conducted as part of this qualitative study. The comparison contextualizes findings from 18 stakeholder interviews (Czech Republic, January 2025-April 2025) against operational governance structures in Austria, Switzerland, the Netherlands, Sweden, and Belgium.

**Table 4.** Comparative overview of affinity domain implementation in selected European countries.

Country	Affinity domain model	Governance structure	National coverage	Key characteristics
Austria	ELGA^a^	Centralized, state-owned	National	Mandatory for providers; opt-out for patients; based on IHE^b^ XDS^c^ [22]
Switzerland	EPD^d^	Federated; regulated at the federal level	Regional or national	Voluntary for patients; multiple affinity domains operated by certified communities [23]
The Netherlands	LSP^e^	Semicentralized	National	Opt-in system national broker connects providers to shared exchange services [24]
Sweden	NPÖ^f^	Regional (county-level)	Regional	Based on regional initiatives, integrated into national strategy and opt-out model [25]
Belgium	Vitalink	Federated (per region)	Regional	Strong focus on authentication via national electronic identity and platform-based document exchange [26]
Czech Republic	Not operational (conceptual phase)	Central coordination planned via MoH^g^ and ÚZIS^h^	None	Legal framework in place, no piloting or operational deployment, and unclear governance

a ELGA: Elektronische Gesundheitsakte

b IHE: Integrating the Healthcare Enterprise.

c XDS: Cross-Enterprise Document Sharing.

d EPD: Elektronisches Patientendossier.

e LSP: Landelijk Schakelpunt.

f NPÖ: Národní plán obnovy.

g MoH: Ministry of Health.

h ÚZIS: Institute of Health Information and Statistics of the Czech Republic.

Comparative analysis illustrates that successful affinity domain models, from Austria’s centralized ELGA to Switzerland’s federated EPD, share common elements: empowered coordinating authorities, integrated legal mandates, robust access infrastructures, and sustained stakeholder engagement. In alignment with interviewees’ accounts of legal uncertainty, fragmented coordination, and the absence of empowered governance bodies, the Czech Republic currently appears to remain at a largely conceptual stage, with limited piloting, incomplete governance clarification, and no operational affinity domain deployment.

The comparison is not intended as an exhaustive review but as an analytical reference illustrating governance configurations that directly contrast with constraints identified in the Czech case. These comparative patterns reinforce the governance gaps identified in the Czech interviews. Our analysis demonstrates that without clearly defined responsibilities, legal clarity, and enforceable standards, practical progress in interoperability remains unattainable. A comparable pattern is observed in a recent World Health Organization (WHO) or European study, which shows that although 83% of countries in the region have adopted a formal digital health strategy, only 79% have fully developed implementation and performance monitoring mechanisms—a relatively high figure, yet with substantial variation in maturity, funding, and institutional authority [27]. A multi-country qualitative study by Papadopoulos et al [18] further underscores that public trust in national EHR systems hinges on data security, privacy, and the perceived legitimacy of actors—key governance issues also relevant for affinity domains. In parallel, a scoping review by Gazzarata et al [14] confirms that although adoption of HL7 FHIR is growing, particularly in chronic disease management, the lack of binding implementation guides, certification frameworks, and coordinating authorities continues to hamper interoperability and data reusability. In affinity domain terms, such structural vendor dependencies undermine enforceable participation rules and coordinated document exchange across institutional boundaries.

Vendor lock-in and proprietary architecture remain structural barriers. As Kapitan et al [28] emphasize, the adoption of open standards such as HL7 FHIR is not sufficient unless accompanied by open-source implementations and integration toolkits tailored to local constraints. A scoping review of European chronic care implementations found that only 20% of projects reference formal implementation guides, and certification or central coordination mechanisms are rare, resulting in persistent heterogeneity that undermines interoperability [14]. A recent review on telemonitoring [29] likewise highlights that despite using FHIR and ISO/IEEE (International Organization for Standardization and the Institute of Electrical and Electronics Engineers) 11073, inconsistent governance models and fragmented implementations remain widespread.

Clinician and stakeholder engagement also remains minimal. This finding directly resonates with interviewees’ descriptions of the limited inclusion of clinical end users in governance processes. According to the Organisation for Economic Co-operation and Development [30], the average quality score for stakeholder engagement in primary lawmaking rose modestly from 2.00 in 2014 to 2.26 in 2024 (on a 0‐4 scale). Engagement in subordinate regulation remains weaker (1.95-2.16), with oversight and quality control scoring particularly low (0.37 out of 1). Despite rapid growth in remote care and patient access metrics, the sustainability of interoperability gains remains uncertain. The Czech Republic’s maturity score increased by 26 points in 2024—the largest annual improvement in the European Union—yet full interoperability remains unachieved, as key structural mechanisms are still absent or only partially implemented.

Building public trust in digital infrastructures is essential. A structured review of semantic interoperability methods shows that meaningful trust-building relies on transparent data standards supported by policy frameworks [31,32]. A cross-country analysis of personal EHR access further validates the importance of standards, usability, and stakeholder inclusion in fostering acceptance and trust [33]. In the absence of such frameworks, Czech narratives around health data governance often neglect data security, privacy, and stakeholder legitimacy—undermining the sustainable implementation of affinity domains.

### Implications for Policy and Practice

The following implications represent author-derived interpretations informed by the empirical findings, relevant literature, and EHDS regulatory requirements, rather than direct requests expressed by the interviewed stakeholders.

Given stakeholders’ repeated emphasis on legal uncertainty, fragmented governance structures, weak enforcement mechanisms, and institutional risk aversion, phased pilot deployments may represent a pragmatic policy instrument for reducing uncertainty and strengthening trust in domain-based interoperability arrangements.

International evidence [22,23,26] suggests that pilots anchored in regional settings and supported by co-design can facilitate stakeholder engagement and accelerate scaling. In the Czech context, and consistent with the governance constraints identified in the interviews, the current absence of pilot deployments, test registries, or structured sandbox environments limits opportunities for iterative validation of both technical and governance arrangements under real-world conditions.

Building on the governance gaps identified in this study, we recommend that the Czech Republic adopt maturity assessment frameworks such as those used in the WHO or European survey [27] or embedded in the EHDS implementation model [34] to evaluate readiness across legal, organizational, and technical dimensions. These tools may guide prioritization, funding allocation, and benchmarking toward operational interoperability.

In alignment with the EHDS regulation, national interoperability governance frameworks—including potential affinity domain structures—should be coordinated with national metadata catalogs and designated health data access bodies to ensure transparency, lawful data access, and structured secondary use.

### Limitations

This study has several limitations. First, purposive and snowball sampling may have over-represented stakeholders who were more accessible or willing to participate, which could limit the transferability of the findings. Second, interviews were conducted with senior policymakers and institutional leaders, including representatives of the MoH and regional authorities. The hierarchical and professional status of these participants may have influenced the framing of responses, potentially introducing elements of social desirability or correctness bias. Although anonymity was guaranteed and reflexive strategies were applied, responses may still reflect institutionally aligned perspectives rather than fully critical accounts. The sample predominantly consisted of highly experienced professionals in senior governance roles. While appropriate for analyzing institutional decision-making and regulatory structures, this composition may under-represent early-career or operational-level perspectives and thus limit generational diversity in the findings. In addition, representatives of primary care professional associations, organized medical chambers, and patient advocacy groups were not directly included. Although senior provider representatives participated, frontline primary care clinicians and organized patient stakeholders were under-represented, which may privilege governance-level perspectives over operational or user-level experiences. Third, while dual independent coding and consensus resolution were implemented, no formal intercoder reliability coefficients were calculated. Fourth, the perspectives analyzed are context-specific to the Czech governance and regulatory environment and may not be generalizable to countries with more mature certification frameworks or long-standing national HIE systems. In addition, the study did not include patients or the general public, despite recurring interview themes related to trust, legitimacy, and institutional credibility. The absence of these perspectives represents a substantive limitation, as public trust is a foundational condition for sustainable affinity domain governance and cross-enterprise data sharing. Furthermore, data collection (January 2025-April 2025) coincided with the finalization and publication of the EHDS regulation in February 2025. The findings may therefore reflect stakeholder perceptions during an early transition phase marked by regulatory uncertainty rather than a stabilized postimplementation environment. Finally, the research did not evaluate pilot implementations or real-world affinity domain deployments, which limits the ability to draw conclusions about operational feasibility. These limitations should be considered when interpreting the results and designing future studies.

### Future Directions

Future research should build on these findings in several directions. First, empirical evaluation of pilot affinity domains is needed to examine how the identified governance attributes—such as role allocation, accountability, and enforcement—operate in practice once interoperability infrastructure moves beyond the conceptual stage. Longitudinal studies following pilot implementations could assess how trust, compliance, and institutional coordination evolve over time.

Second, future research should prioritize the inclusion of patients and public representatives, particularly to examine how trust, legitimacy, and perceived data governance affect the willingness to participate in affinity domain–based information exchange. Incorporating these perspectives would allow for the triangulation of governance assumptions identified in this study.

Third, comparative research across post-transition health systems could help distinguish context-specific barriers from more general governance patterns affecting affinity domain implementation. Such work would also support benchmarking national readiness in relation to EHDS requirements and inform transferable policy lessons.

### Conclusions

This study examined stakeholder perceptions of affinity domains as a governance and interoperability construct within the Czech health care system using qualitative interviews. The findings show that while affinity domains are recognized as a promising model for cross-enterprise data sharing, their feasibility is constrained by unclear governance mandates, limited enforcement mechanisms, legal uncertainty, and fragmented implementation capacity. By explicitly linking these challenges to the governance attributes of affinity domains, the study clarifies why interoperability barriers persist despite the availability of technical standards. These findings contribute empirical insight into the governance conditions required for operational interoperability in post-transition health systems and provide a grounded basis for further policy and implementation research.

## Supplementary material

10.2196/83894 Multimedia Appendix 1 Participant characteristics.

10.2196/83894 Multimedia Appendix 2 Interview guide.

10.2196/83894 Multimedia Appendix 3 Saturation grid.

10.2196/83894 Multimedia Appendix 4 Coding tree and exemplar codes.

10.2196/83894 Multimedia Appendix 5 Extended codebook with illustrative quotes.

10.2196/83894 Checklist 1 COREQ checklist.

## Abbreviations

COREQ: Consolidated Criteria for Reporting Qualitative Research
DICOM: Digital Imaging and Communications in Medicine
EHDS: European Health Data Space
EHR: electronic health record
ELGA: Elektronische Gesundheitsakte
eMeDOcS: Exchange Medical Documents System
EPD: Elektronisches Patientendossier
FHIR: Fast Healthcare Interoperability Resources
HIE: health information exchange
HIF: health insurance fund
HL7: Health Level 7
IEEE: Institute of Electrical and Electronics Engineers
IHE: Integrating the Healthcare Enterprise
ISAC: Integration, Share and Communication System
ISO: International Organization for Standardization
IZIP: Internetový zdravotní informační portál
MoH: Ministry of Health
RHA: regional health authority
WHO: World Health Organization
XDS: Cross-Enterprise Document Sharing
