# Supplementary material for: Stakeholder Perspectives on Affinity Domains in Digital Health Interoperability: Qualitative Study
Source: JMIR Med Inform. 2026 Apr 2;14:e83894. doi: 10.2196/83894 (PMC13046094; doi:10.2196/83894)
Supplement: Multimedia Appendix 1 [file medinform-v14-e83894-s001.docx]

**Participant Characteristics**

| **Stakeholder group** | **n** | **Sector** | **Region (national/regional)** | **Years of professional experience*** |
| --- | --- | --- | --- | --- |
| Ministry of Health (MoH) | 3 | Public | National | 10, 12, 15 |
| Regional Health Authorities (RHA) | 3 | Public | Regional | 8, 10, 12 |
| Healthcare Providers (HP) | 4 | Mixed (public/private) | Regional/national hospitals | 15 (12–20) |
| Health Insurance Funds (HIF) | 2 | Public | National | 12, 18 |
| Health IT Vendors (HV) | 4 | Private | National / regional | 11 (9–14) |
| Independent Consultants and Expert Advisors (ICEA) | 2 | Private/independent | National | 15, 22 |
| **Total** | **18** | — | — | — |

*For groups with n=4, values are presented as median (interquartile range); for groups with n≤3, raw values are reported.
