# Supplementary material for: Stakeholder Perspectives on Affinity Domains in Digital Health Interoperability: Qualitative Study
Source: JMIR Med Inform. 2026 Apr 2;14:e83894. doi: 10.2196/83894 (PMC13046094; doi:10.2196/83894)
Supplement: Multimedia Appendix 2 [file medinform-v14-e83894-s002.docx]

**Semi-Structured Interview Guide**

1. **Introductory Standardised Explanation**

At the beginning of each interview, participants were provided with a brief standardised explanation of the concept of an “affinity domain” based on the IHE XDS framework. The explanation emphasised:

- shared governance rules,
- defined membership criteria,
- accountability and liability arrangements,
- enforcement mechanisms,
- cross-enterprise document sharing under agreed interoperability standards (IHE XDS, HL7 FHIR).

Participants were asked to reflect on feasibility, prerequisites, risks, and governance conditions specifically in relation to this defined model.

1. **Thematic Structure of the Interview Guide**

The interview guide followed four core domains. Questions were open-ended. Probing questions were used flexibly to clarify, deepen, or exemplify responses. The guide was piloted with two external experts and slightly refined for clarity and flow.

**Domain 1: Governance Roles and Responsibilities**

1. How do you perceive the current distribution of roles and responsibilities in Czech digital health governance?
2. Who should hold rule-making authority in an affinity domain model?
3. What institution should be responsible for coordination and enforcement?
4. How should accountability for shared health data be allocated?

Probing examples:

- Can you give a concrete example?
- What would happen if no central authority existed?
- How does this compare to current practice?

**Domain 2: Legal and Regulatory Conditions**

1. Are current legal frameworks sufficient for implementing affinity domains?
2. How should liability be handled when data are shared across institutions?
3. What regulatory changes would be necessary?

Probing examples:

- Who bears responsibility if data are incomplete or outdated?
- Are consent mechanisms sufficiently defined?
- Where do you see legal ambiguity?

**Domain 3: Technical Readiness and Standards**

1. How do you assess current interoperability readiness in Czech healthcare?
2. Are IHE XDS and HL7 FHIR sufficiently adopted?
3. What technical barriers could prevent affinity domain implementation?
4. Should interoperability compliance be voluntary or mandatory?

Probing examples:

- What role do vendors currently play?
- Is certification necessary?
- What would ensure enforceability?

**Domain 4: Risks, Benefits, and Feasibility**

1. What risks do you associate with implementing affinity domains?
2. What benefits could realistically be achieved?
3. What conditions must be fulfilled before implementation?
4. Would pilot deployments be feasible?

Probing examples:

- How would clinicians respond?
- How would financing be handled?
- What would increase stakeholder trust?
