# Supplementary material for: Stakeholder Perspectives on Affinity Domains in Digital Health Interoperability: Qualitative Study
Source: JMIR Med Inform. 2026 Apr 2;14:e83894. doi: 10.2196/83894 (PMC13046094; doi:10.2196/83894)
Supplement: Multimedia Appendix 3 [file medinform-v14-e83894-s003.docx]

**Saturation Grid**

| Interview nr. | New codes identified? | Examples of new codes (in vivo) | Notes on saturation |
| --- | --- | --- | --- |
| 1 | Yes | “No one enforces the strategy”, “isolated islands” | Early governance/fragmentation themes |
| 2 | Yes | “vendors dictate what’s possible” | Vendor dominance |
| 3 | Yes | “No national integration in sight” | Confirms fragmentation |
| 4 | Yes | “IZIP left a scar” | Institutional distrust |
| 5 | Yes | “No technical backbone” | Technical inconsistency |
| 6 | Yes | “Who takes responsibility?” | Legal uncertainty |
| 7 | Yes | “Doctors not consulted” | Clinical exclusion |
| 8 | Yes | “CT scans repeated twice” | Redundancy/cost |
| 9 | Yes | “another bureaucratic project” | Trust/engagement barrier |
| 10 | Yes | “No one checks interoperability” | Confirms fragmentation |
| 11 | Yes | “Who pays for changes?” | Funding/incentives |
| 12 | Yes | “The biggest bottleneck is people” | HR capacity → **last new code** |
| 13–18 | No | – | No new major themes \| Minor variations within existing categories \| Thematic saturation confirmed at the level of higher-order themes; subsequent interviews reinforced and deepened existing categories without generating conceptually distinct codes. |
