# Supplementary material for: Stakeholder Perspectives on Affinity Domains in Digital Health Interoperability: Qualitative Study
Source: JMIR Med Inform. 2026 Apr 2;14:e83894. doi: 10.2196/83894 (PMC13046094; doi:10.2196/83894)
Supplement: Multimedia Appendix 4 [file medinform-v14-e83894-s004.docx]

**Coding tree & exemplar codes**

The major categories were derived inductively. For analytical clarity, each category was subsequently aligned with governance dimensions constitutive of affinity domains, as specified in Table 2 in main text.

| **Major category (Inductive)** | **Subthemes** | **Affinity domain dimension**  **(Table 2)** |
| --- | --- | --- |
| **Roles and responsibilities** | Ambiguity of governance roles; Vendor dominance; Regional fragmentation; Clinical end-user exclusion | Shared rule-making; Membership and participation |
| **Perceived risks** | Institutional distrust (IZIP legacy); Legal uncertainty; Technical fragmentation; Vendor lock-in | Trust relationships; Accountability and liability; Enforcement mechanisms; Technical scope (IHE XDS) |
| **System-level prerequisites** | Independent governance body; Funding and incentives; Standards adoption; Inclusion of social care; Human resource constraints | Shared rule-making; Membership and participation; Enforcement mechanisms; Technical scope (IHE XDS) |
| **Perceived benefits and opportunities** | Improved coordination; Reduced redundancy; Continuity of care; Accountability and secondary use | Accountability and liability; Trust relationships; Membership and participation; Technical scope (IHE XDS) |
| **Implementation barriers** | Legal uncertainty; Political discontinuity; Vendor resistance; Limited stakeholder engagement | Shared rule-making; Enforcement mechanisms; Membership and participation; Trust relationships |

Note: The coding structure reflects empirically derived themes, which were subsequently interpretively mapped onto the predefined affinity domain governance dimensions specified in Table 2 to ensure analytical consistency with the theoretical framework.
