# Supplementary material for: Stakeholder Perspectives on Affinity Domains in Digital Health Interoperability: Qualitative Study
Source: JMIR Med Inform. 2026 Apr 2;14:e83894. doi: 10.2196/83894 (PMC13046094; doi:10.2196/83894)
Supplement: Multimedia Appendix 5 [file medinform-v14-e83894-s005.docx]

**Extended codebook with illustrative quotes**

**Theme A: Roles and Responsibilities**

- Ambiguity of governance roles → “The Ministry sets strategies but does not enforce them.” (R18, ICEA)
- Regional fragmentation → “Each region develops its own system…” (R4, RHA)
- Vendor dominance → “In some hospitals, the vendor decides more than the IT department. They lock the system and then dictate what’s possible.” (R13, HV).
- Clinical end-user exclusion → “We receive new systems and tools, but no one asked whether they match how we work. That’s why so many doctors ignore them.” (R7, HP).

**Theme B: Perceived Risks**

- Institutional distrust → “IZIP left a scar.” (R17, ICEA)
- Legal uncertainty → “If a patient is harmed…” (R8, HP)
- Technical fragmentation → “No technical backbone…” (R14, HV)
- Vendor lock-in → “Why would a vendor enable sharing…” (R15, HV)

**Theme C: System-level Prerequisites**

- Independent governance body → “Someone has to own it.” (R2, MoH)
- Funding and incentives → “Who pays for changes?” (R11, HIF)
- Inclusion of social care → “Social care system is half the equation.” (R5, RHA)
- Human resources → “Bottleneck is people who understand standards.” (R18, ICEA)

**Theme D: Perceived Benefits**

- Reduced redundancy → “Same CT scans repeated.” (R8, HP)
- Continuity of care → “GP shouldn’t guess after discharge.” (R7, HP)
- Accountability → “Audit trails help trace responsibility.” (R1, MoH)

**Theme E: Implementation Barriers**

- Legal uncertainty → “We operate in grey zones.” (R6, RHA)
- Political discontinuity → “One government cancels what the last one started.” (R18, ICEA)
- Vendor resistance → “If not in contract, vendors don’t care.” (R15, HV)
- Limited stakeholder engagement → “Doctors hear about projects only at rollout.” (R9, HP)
