# Supplementary material for: Stakeholder Perspectives on Affinity Domains in Digital Health Interoperability: Qualitative Study
Source: JMIR Med Inform. 2026 Apr 2;14:e83894. doi: 10.2196/83894 (PMC13046094; doi:10.2196/83894)
Supplement: Checklist 1 [file medinform-v14-e83894-s006.docx]

# COREQ Checklist

## Domain 1: Research team and reflexivity

### Interviewer/facilitator (Item 1)

Guide question: Which author/s conducted the interview or focus group?

Reported in manuscript: Methods – Interviews conducted by PH and IF.

### Credentials (Item 2)

Guide question: What were the researcher’s credentials? E.g. PhD, MD

Reported in manuscript: Methods – Researchers’ credentials described (Ph.D., Ing., assoc. prof.).

### Occupation (Item 3)

Guide question: What was their occupation at the time of the study?

Reported in manuscript: Methods – Occupations reported (researchers at FBMI CTU and TUL).

### Gender (Item 4)

Guide question: Was the researcher male or female?

Reported in manuscript: Methods – Gender specified (PH, IF women; JB man).

### Experience and training (Item 5)

Guide question: What experience or training did the researcher have?

Reported in manuscript: Methods – Training and prior experience in qualitative health research described.

### Relationship established (Item 6)

Guide question: Was a relationship established prior to study commencement?

Reported in manuscript: Methods – Brief professional contacts via networks, no dependency.

### Participant knowledge of the interviewer (Item 7)

Guide question: What did the participants know about the researcher?

Reported in manuscript: Methods – Participants informed about affiliation, aims, and interests.

### Interviewer characteristics (Item 8)

Guide question: What characteristics were reported about the interviewer/facilitator?

Reported in manuscript: Methods – Bias mitigation described (audit trail, dual coding, reflexive memos).

## Domain 2: Study design

### Methodological orientation and theory (Item 9)

Guide question: What methodological orientation was stated to underpin the study?

Reported in manuscript: Methods – Qualitative, exploratory, thematic analysis, COREQ framework.

### Sampling (Item 10)

Guide question: How were participants selected?

Reported in manuscript: Methods – Purposive and snowball sampling.

### Method of approach (Item 11)

Guide question: How were participants approached?

Reported in manuscript: Methods – Email invitations and professional networks.

### Sample size (Item 12)

Guide question: How many participants were in the study?

Reported in manuscript: Methods – 18 participants.

### Non-participation (Item 13)

Guide question: How many people refused to participate or dropped out? Reasons?

Reported in manuscript: Methods – 22 invited, 4 declined due to time constraints.

### Setting of data collection (Item 14)

Guide question: Where was the data collected?

Reported in manuscript: Methods – Online secure video-conferencing.

### Presence of non-participants (Item 15)

Guide question: Was anyone else present besides the participants and researchers?

Reported in manuscript: Methods – Only interviewer and participant.

### Description of sample (Item 16)

Guide question: What are the important characteristics of the sample?

Reported in manuscript: Methods + Appendix 1 – Stakeholder groups and roles.

### Interview guide (Item 17)

Guide question: Were questions, prompts, guides provided by the authors? Was it pilot tested?

Reported in manuscript: Methods – Guide developed from literature and pilot-tested with 2 experts.

### Repeat interviews (Item 18)

Guide question: Were repeat interviews carried out? If yes, how many?

Reported in manuscript: Methods – No repeat interviews conducted.

### Audio/visual recording (Item 19)

Guide question: Did the research use audio or visual recording to collect the data?

Reported in manuscript: Methods – All interviews audio-recorded with consent.

### Field notes (Item 20)

Guide question: Were field notes made during and/or after the interview or focus group?

Reported in manuscript: Methods – Detailed field notes made after sessions.

### Duration (Item 21)

Guide question: What was the duration of the interviews or focus group?

Reported in manuscript: Methods – Interviews lasted approximately 50 minutes.

### Data saturation (Item 22)

Guide question: Was data saturation discussed?

Reported in manuscript: Methods + Appendix 2 – Saturation after 12 interviews; saturation grid provided.

### Transcripts returned (Item 23)

Guide question: Were transcripts returned to participants for comment and/or correction?

Reported in manuscript: Methods – Transcripts returned to participants for member checking.

## Domain 3: Analysis and findings

### Number of data coders (Item 24)

Guide question: How many data coders coded the data?

Reported in manuscript: Methods – Two coders (PH, IF), consensus resolution.

### Description of the coding tree (Item 25)

Guide question: Did authors provide a description of the coding tree?

Reported in manuscript: Methods + Appendix 3 – Coding tree provided.

### Derivation of themes (Item 26)

Guide question: Were themes identified in advance or derived from the data?

Reported in manuscript: Methods – Themes derived inductively from data.

### Software (Item 27)

Guide question: What software, if applicable, was used to manage the data?

Reported in manuscript: Methods – MAXQDA 24 used for data management and analysis.

### Participant checking (Item 28)

Guide question: Did participants provide feedback on the findings?

Reported in manuscript: Methods – Member checking performed; clarifications incorporated.

### Quotations presented (Item 29)

Guide question: Were participant quotations presented to illustrate the themes/findings?

Reported in manuscript: Results – Quotes labelled with respondent code (R#) and group.

### Data and findings consistent (Item 30)

Guide question: Was there consistency between the data presented and the findings?

Reported in manuscript: Results + Discussion – Findings supported by presented data and quotations.

### Clarity of major themes (Item 31)

Guide question: Were major themes clearly presented in the findings?

Reported in manuscript: Results – Five major categories clearly presented.

### Clarity of minor themes (Item 32)

Guide question: Is there a description of diverse cases or discussion of minor themes?

Reported in manuscript: Results – Where present, minority or divergent perspectives were noted; however, the findings predominantly reflected cross-stakeholder consensus.
